# Forecast attribution reveals enhanced heat mortality from climate change in British Columbia heatwave

**DOI:** 10.1126/sciadv.adw8268

**Published:** 2025-11-19

**Authors:** Chin Yang Shapland, Y. T. Eunice Lo, Nicholas J. Leach, Éric Lavigne, Kate Tilling, Dann M. Mitchell

**Affiliations:** ^1^ MRC Integrative Epidemiology Unit at the University of Bristol, Bristol, UK.; ^2^ Population Health Sciences at the University of Bristol, Bristol, UK.; ^3^ Cabot Institute for the Environment, University of Bristol, Bristol, UK.; ^4^ School of Geographical Sciences, University of Bristol, Bristol, UK.; ^5^ Elizabeth Blackwell Institute for Health Research, University of Bristol, Bristol, UK.; ^6^ Atmospheric, Oceanic, and Planetary Physics, Department of Physics, University of Oxford, Oxford OX1 3PU, UK.; ^7^ School of Epidemiology & Public Health, Faculty of Medicine, University of Ottawa, Ottawa, Canada.; ^8^ Environmental Health Science and Research Bureau, Health Canada, Ottawa, Canada.

## Abstract

In 2021, Canada experienced one of the most extreme heatwaves ever seen anywhere on the globe. We use a weather forecast model to attribute health impacts to climate change. We simulate the heatwave as a present-day forecast, a preindustrial-counterfactual scenario, and a future-counterfactual scenario. Despite the extremeness of the event, our analysis shows that, under current climate conditions, we could have still seen up to 30% more heat-related deaths than the number observed. We show that between 11 and 15% of the observed human mortality was attributable to climate change during this event, depending on the conditioning of the atmospheric circulation. We also show that, had “the same event” occurred in the future, the additional mortality toll is between 5 to 18%. We argue that this method gives particularly reliable impact attribution results and is therefore strongly defensible in decision-making and legal settings.

## Teaser

Forecast attribution shows the unseen mortality extremes during British Columbia 2021 heatwave and shifts due to climate change.

## INTRODUCTION

The exposure to extreme and high temperature episodes has been shown to be associated with adverse human health (*1*). The immediate health outcomes include mortality, hospital admittance, visits to the intensive care unit, mental health issues, and adverse pregnancy and birth outcomes (*2*, *3*), but there is also a longer-term health burden due to persistent exposures (*4*). Because of anthropogenic climate change, extreme heat events are becoming more frequent in nearly every location of the globe, and many communities are being caught “off guard” (*5*). One of the most extreme heatwaves in observational records occurred between 28 and 30 June 2021 (*6*), where the Pacific Northwest (PNW) experienced a record-breaking temperature of 49.6°C. The local coroner’s report estimated that 619 heat-related deaths occurred during this event (*7*), with an increase in emergency department visits (*8*). British Columbia Coroners also noted increased severe mental illness and substance use occurring between 25 June and 1 July 2021 (*7*).

Quantifying the health impact attributable to anthropogenic greenhouse gas emissions has played an essential role toward informing policy-makers of adaptation and mitigation (*9*) and in climate litigation (*10*). Now, there are nine studies that undertook a climate change attribution of a hazard to a health outcome (*11*). They estimated that the climate-attributable mortality was 20, 27, and 69% during the summer heatwave in London (2006), Zürich (2018), and Paris (2003), respectively (*12*, *13*). A total of 271,656 heat-related deaths were estimated across 43 countries, averaging over summers of 1991 to 2018 (*14*). The latest study estimated that 56% [95% confidence interval (CI): 39 and 77%] of deaths during the 2022 summer in Europe was due to anthropogenic warming (*15*). These studies first calculated the relationship between mortality and daily temperatures from observed data and then inferred anthropogenic climate change attributable deaths with temperatures from a counterfactual scenario where climate was induced by natural forcings only.

The temperatures observed in the PNW heatwave 2021 have been deemed “impossible” in previous papers from conventional statistical models in attribution (*5*, *16*–*18*). This has been ascribed to the exceptional meteorological conditions that led up to the event (the PNW heatwave was driven by a particular set of optimal physical drivers, which had not appeared previously in the historical record). Studies since have shown that using more complex statistical or dynamical approaches that explicitly include the impact of these drivers can mitigate this “impossibility” issue (*19*). Despite the unprecedented nature of the event within the context of the historical record, weather forecast models were able to predict this extreme heatwave (*20*). Their ability to capture the necessary processes involved suggested that they would be suitable for use within a forecast-based approach to extreme event attribution (*21*), rather than using coarser resolution climate models. This was done by perturbing the boundary and initial conditions in operational forecast models to simulate different counterfactual scenarios, with “preindustrial” involving the removal of anthropogenic influence from the ocean state and reducing atmospheric carbon dioxide (CO_2_), and “future” involving the same perturbation but in the opposite direction. Using this technique, previous research showed that the PNW heatwave was made at least eight times more likely by anthropogenic climate change (*21*).

Attribution statements of impacts need to be reliable, and in general, the more chaotic the part of the climate system that is involved, the harder it is to achieve this reliability. Impacts, in particular, are often felt at local spatial scales, often kilometers, or tens of kilometers, where climate models can have very poor skill. One method to sensibly undertake attribution analyses of these complex processes at fine spatial scales is by using weather forecast models, but run in counterfactual climates. Such weather models are routinely assessed for their reliability. They provide a step change in improvement against using climate models for attribution assessments. Furthermore, weather forecast models are often much less biased than climate models (in part due to their initialization); hence, the statistical bias correction approaches typically used in climate model–based impact studies are not required. These approaches to bias correction increase the amount of epistemic uncertainty and can modify attributable climate responses, thus making bias correction–free approaches attractive. We have included accessible definition of “climate” and “weather” in Supplementary Text.

Having attribution statements made from weather forecast models at local spatial scales is also an important advantage for health service planning. Local health services are not always well funded; in areas most likely affected by heatwaves, the local health services could claim more financial support from central funding using such statements. Moreover, this forecast-based framework of providing probabilities and heat-related death counts at different lead times is particularly advantageous in preparing local health services. For example, health services would know that a 3-day lead time at “future” scenario is the most accurate but with little time to plan for potential mortality impact at a given probability, and an 11-day lead time will give them the least accurate prediction but with more time to plan ahead. The balancing of risks in health services is common practice, i.e., the UK National Institute for Health and Care Excellence is constantly deciding on which drug gets introduced to the UK health services by balancing effectiveness of the drug and its cost. By offering these quantitative decision parameters, health services will have another tool in preparation for future heatwave events. Relatedly, as weather models are already run routinely at weather forecast centers, operational pipelines for translating “ahead-of-time” attribution to adverse health outcomes could be incorporated.

This impact attribution study uses an operational weather forecast model, rather than a climate model, to consider the health impact of the PNW heatwave that is attributable to human influence on the climate. The aim of this paper is to provide a reliable estimate of heat-related deaths under “preindustrial,” “current,” and “future” scenarios, derived from the forecast-based approach to extreme event attribution.

## RESULTS

We use the Integrated Forecasting System (IFS) model from the European Centre for Medium-Range Weather Forecasts (ECMWF) to simulate the PNW heatwave, at 18-km spatial resolution. The operational version of IFS at the time of the event was demonstrably able to simulate the physical processes necessary to forecast the PNW heatwave, despite its unprecedented nature within the context of the historical record (*20*–*23*). The ensemble forecast from the ECMWF suggested that a heatwave of comparable magnitude was plausible at lead times of around 10 days.

We choose three different initialization times for the model to reflect a highly conditioned attribution approach, not dissimilar to the storyline approach [e.g., Zappa and Shepherd (*24*)], all the way through to an unconditioned approach, more similar to large-ensemble climate modeling [e.g., King *et al.* (*25*) and Massey *et al.* (*26*)]. These three lead times end in the heatwave peak period of 2021 (28 to 30 June) and occur 3, 7, and 11 days before this. One way of viewing these three attribution experiments is that the 3-day lead time is highly conditioned on the heat dome dynamics of the event, but it allows the thermodynamics to vary. Such an approach asks, “Given changes in anthropogenic emissions, but keeping the circulation very similar, how do characteristics of the event change?” Whereas the 11-day lead time does not constrain the dynamics because they were not particularly predictable on those temporal scales, and therefore has a range of different dynamical regimes along with the thermodynamical changes. This approach asks, “Given changes in anthropogenic emissions, how do characteristics of the event change?” Three- and 7-day lead times have a 51-member ensemble, and an 11-day lead time has a 251-member ensemble. See more details in the Materials and Methods section.

### Temperature response in the climate simulations

**Fig. 1.**
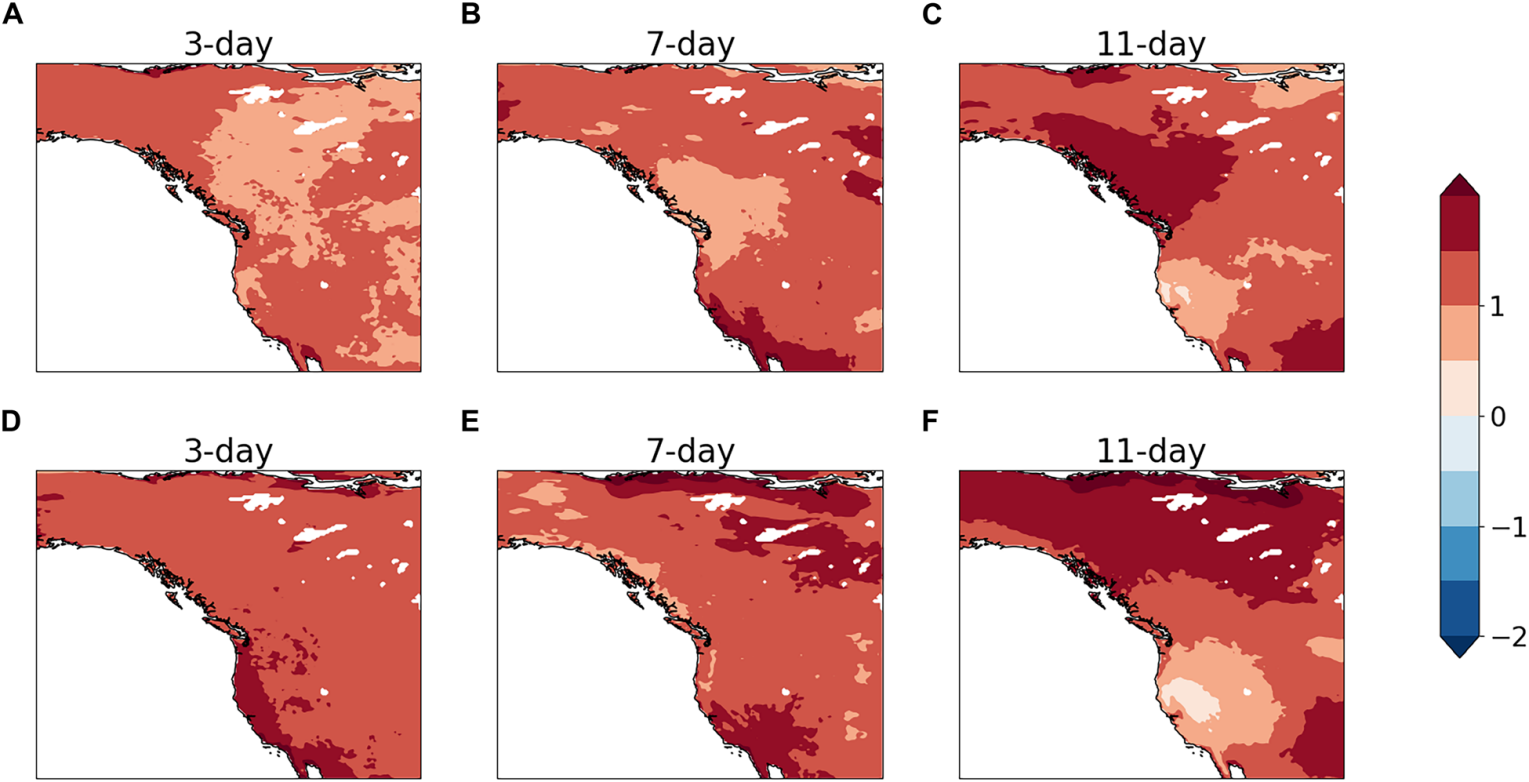
Difference in daily average temperature between current and preindustrial and between future and current of the PNW region (−150°E to −100°E longitude, 70°N to 30°N latitude). (**A** to **C**) Difference between current and preindustrial for forecast lead times 3, 7, and 11 days, respectively. (**D** to **F**) Difference between future and current for forecast lead times 3, 7, and 11 days, respectively. Average temperature during 26 to 30 June 2021. Averaged over a 51-member ensemble for 3 and 7 days and a 251-member ensemble for 11 days.

Figure 1 (A to C) shows that the forecasted averaged near-surface air temperature with current climate conditions is hotter than temperatures in preindustrial conditions. This is true for all three lead times. The PNW region (−150°E to −100°E longitude, 70°N to 30°N latitude) in the current climate is, on average, 1.2°, 1.3°, and 1.4°C warmer than preindustrial climate at 3-, 7-, and 11-day lead times, respectively. The same metric, but comparing the future and current climate, is, on average, 1.4°, 1.3°, and 1.4°C, respectively, hotter under the future climate conditions than with current climate conditions (Fig. 1, D to F). This indicates that, although the global mean warming levels are equivalent in the top and bottom panels, there are regional differences.

### Converting the hazard into human mortality

It is important that not only the hazard is reliably captured but also the impact of that hazard. Although the hazard has been assessed in the precursor papers (*21*, *27*), the mortality model has not yet been validated. Table 1 gives the summary of the observed all-cause mortality and daily mean temperature (°C) during the summer months of 1981 to 2015 in four health service delivery areas (HSDAs) that contained the four major cities (Abbotsford, Lytton, Vancouver, and Victoria) that were most affected by the 2021 PNW heatwave. See more details in the Materials and Methods section.

**Table 1.** Summary of the observed temperature and all-cause mortality over the summer months (June to September) of 1981-2015 in HSDAs. SD, standard deviation.

HSDA	Data period	Total deaths	Mean daily deaths (SD)	Mean daily average temperature (SD)
Fraser East	1981–2015	18,275	4.28 (2.33)	16.7 (3.17)
Thompson/Cariboo	1981–2015	15,334	3.59 (2.13)	15.1 (3.74)
Vancouver	1991–2007	67,818	32.7 (6.17)	16.8 (2.61)
Vancouver*	2008–2015	9,375	9.61 (3.18)	17.4 (2.75)
South Vancouver Island	1981–2015	34,423	8.06 (2.94)	15.7 (2.35)

* The health boundaries of Vancouver have changed in 2008.

The British Columbia Coroner’s report (*7*) has shown a number of heat-related deaths by Townships and Health Authority over 25 June to 1 July. Health Authority covers several HSDAs, for example, Vancouver Coastal Health Authority covers Richmond, Vancouver, and North Shore/Coast Garibaldi HSDAs. Potentially, the township of Vancouver may be comparable to Vancouver HSDA, although the report was not clear about the geographical boundaries of Townships. The number of heat-related deaths in the township of Vancouver and Vancouver Coastal Health Authority was 117 and 145, respectively. We will preempt here that the epidemiological model will not be able to estimate the same value of heat-related deaths as the model calculates heat-related deaths from expected daily deaths, which, from Table 1, is averaged as 9.6 deaths from summers up until year 2015, and the temperature for heatwave attribution is between 26 and 30 June 2021.

**Fig. 2.**
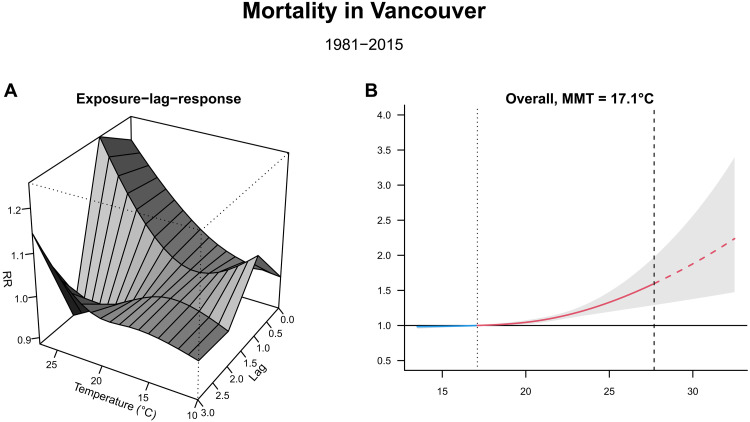
Temperature-related mortality in Vancouver (1991 to 2015). (**A**) Three-dimensional plot showing the estimated exposure-lag-response association between temperature and mortality, where the *y* axis, *x* axis, and *z* axis are the RR, daily average temperature (in °C), and lag time (in days), respectively. (**B**) Plot showing the overall cumulative mortality risk; *y* axis is the RR, and the *x* axis is the daily average temperature in degrees Celsius. The dotted line is the minimum mortality temperature (MMT). The dashed part of the curve is the extrapolation beyond the maximum temperature observed in 1981 to 2015 (dashed vertical line).

Our model shows that, in Vancouver HSDA, the relative risk (RR) of mortality increases with daily average temperature (Fig. 2B). The minimum mortality temperature is at 17.1°C, the RR exponentially increases to 1.60 (95% CI: 1.34 to 1.91) when the daily average temperature is risen to 27°C (hottest daily average observed). At hottest temperatures, the RR drops after 1-day lag (after heat exposure), showing that high temperatures have an immediate effect on all-cause mortality (Fig. 2A). Fraser East HSDA (containing Abbotsford) also shows an increase in RR of mortality with rising temperature (fig. S1). There is no evidence of an effect of temperature on mortality in Thompson/Cariboo or South Vancouver Island HSDA (figs. S2 and S3, respectively). This could be due to rare all-cause mortality events and extreme heat events during 1981 to 2015 as the model accounts for variation between years and fits nonlinear effects, resulting in the wide CI. Alternatively, there could be genuine differences in effects of temperature on all-cause mortality between HSDAs, for example, due to urban heat islands, age structure, or proximity to a health care facility.

For sensitivity analysis, we investigated whether the risk is different between genders and age categories. In Vancouver HSDA, when data are stratified by under age 65, between ages 65 and 74, and over age 74, there is no evidence of difference between the three subgroups in the nonlinear relationship of average temperature and mortality (fig. S4A). There is a difference in minimum mortality temperature, 19.4° and 17.1°C, for under 65 and over 74 age categories, respectively. We did not find evidence of different effects of temperature on mortality between female and male (fig. S4B). It is important to note that no evidence of an effect does not mean there is no effect, and as demonstrated by the wide CIs, our sensitivity analyses are underpowered; once all-cause mortality is stratified, the number of events is rarer in the summertime of each year and our epidemiological model has many degrees of freedom as it accounts for seasonal effects (see the Materials and Methods section). There is also no evidence of a difference between subgroups in other HSDAs (figs. S5 to S7).

### Climate attribution of heat mortality at different lead times

**Fig. 3.**
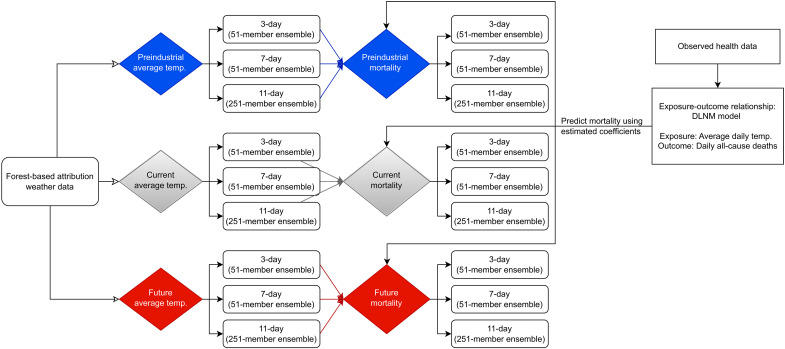
Framework summary. Summary to illustrate the translation of forest-based attribution of temperature into heat mortality.

For the HSDA that showed association between daily average temperature and all-cause mortality, we extracted the daily average temperatures from the nearest latitude and longitude coordinates from the gridded forecast-based climate simulations. We then extrapolated the RR of these predicted temperatures from the temperature-mortality curve of all ages and gender (e.g., red dashed line in Fig. 2B). Last, we applied these RRs to the daily expected deaths to predict the number of heat-related deaths. We have summarized these steps in Fig. 3, and more comprehensive details are in the Materials and Methods section. Here, we will give a full description of the results in Vancouver HSDA and a summary of the results for Fraser East HSDA.

#### Vancouver results

**Fig. 4.**
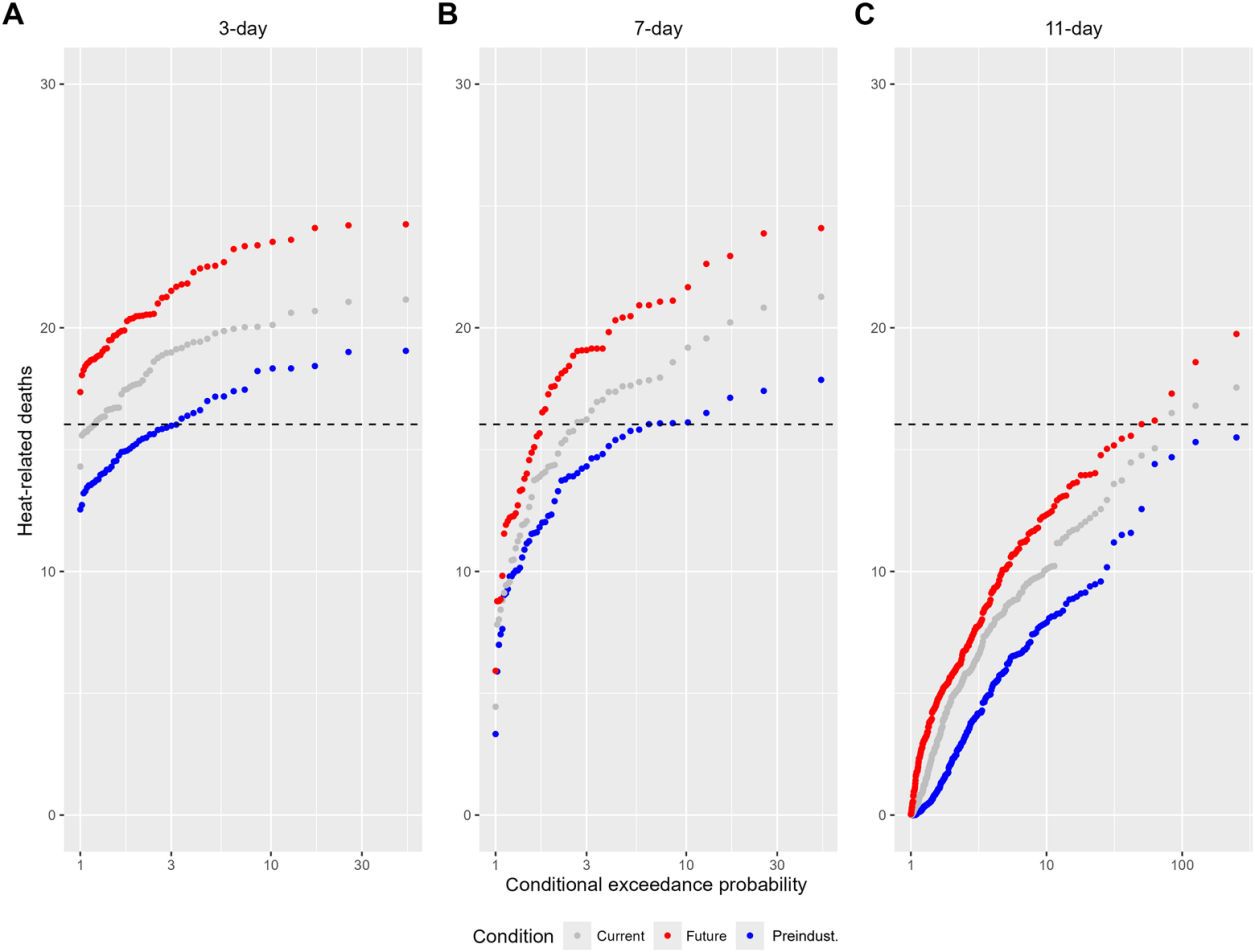
Conditional exceedance probability for heat-related deaths during the PNW heatwave predicted from operational and counterfactual forecast ensembles in Vancouver. The *y* axis is the sum of heat-related deaths across 26 to 30 of June 2021 (i.e., PNW heatwave). The *x* axis is log scaled. The conditional exceedance probability of ensemble member *i* is calculated as the total number of ensemble members divided by rank (-1*sum of deaths over the heatwave period of *i*). (**A** to **C**) shows ensembles initialized at lead times 3, 7, and 11 days respectively, as shown above each panel. Red, gray, and blue dots indicate empirical return-time plots for heat-related mortality predicted from daily mean temperatures of the ensemble members of the future, current, and preindustrial forecasts. The black dashed line shows heat-related deaths based on the observed average temperature (ERA5 reanalysis) during the PNW heatwave.

Figure 4 gives the conditional exceedance probability of heat-related deaths during the PNW heatwave event (sum of deaths within 26 to 30 June 2021), showing that “future” conditions (colored red) are very likely to have higher heat-related mortality in comparison to “current” and “preindustrial” conditions (colored gray and blue, respectively). The conditional exceedance probability is the probability of the same observed event reoccurring or exceeding the observed event, conditional on the initial state of the forecast model (see the Materials and Methods section for more detail). In short, the greater the conditional exceedance probability, the less likely the event would occur. The dashed line in Fig. 4 gives the heat-related deaths counts (= 16) based on the observed daily average temperature [ERA5 reanalysis (*28*)] for Vancouver HSDA during the heatwave event.

To contextualize the impact of the heatwave event, we express impact as the heat-attributable fraction of mortality, calculated as the predicted heat-related deaths during the heatwave event divided by the corresponding sum of expected daily all-cause deaths. For example, the heat-attributable fraction of mortality is 34% (= 16/47) in Vancouver because there were 16 observed heat-related deaths and 47 expected all-cause deaths in the same time period.

As the lead time to the date of the PNW heatwave event increases (going from Fig. 4, A to C), the probability of observing the same heat-related mortality (the dashed line) reduces. This is due to the reduced level of conditioning on the dynamics of the weather system at longer lead times. It is particularly useful to compare the 3- and 11-day lead times (Fig. 4, A and C) because they give us very different attribution statements. This is not unexpected, given the framing of each question (see Introduction). For the 3-day lead time, the Earth system state that is driving the heat event is already extreme. For instance, the atmospheric flow is highly constrained to be extreme, so most ensemble members reflect the corresponding high temperatures. For the 11-day forecast, the atmospheric conditions have evolved into many less extreme states, so the corresponding temperatures reflect that (*27*).

#### Three-day lead time

When the forecast model is initialized 3 days before the heatwave event, most of the ensemble members have predicted the same number of heat-related deaths as the observed temperatures (dashed line in Fig. 4) for the “current” scenario (gray dots in Fig. 4A). Under the “future” climate condition (red dots), it is unlikely to observe the same number of heat-related deaths as the PNW heatwave; it will be higher than observed. For the “preindustrial” scenario (blue dots), on the day of the heatwave forecast initialization, there was a 1-in-3.2 (conditional exceedance probability) chance that this mortality could have been reached or exceeded the observed value (dashed line in Fig. 4). In other words, if a near-identical circulation pattern and synoptic setup were experienced in a preindustrial climate, there is a 1-in-3.2-year chance of repeating the same as observed.

**Fig. 5.**
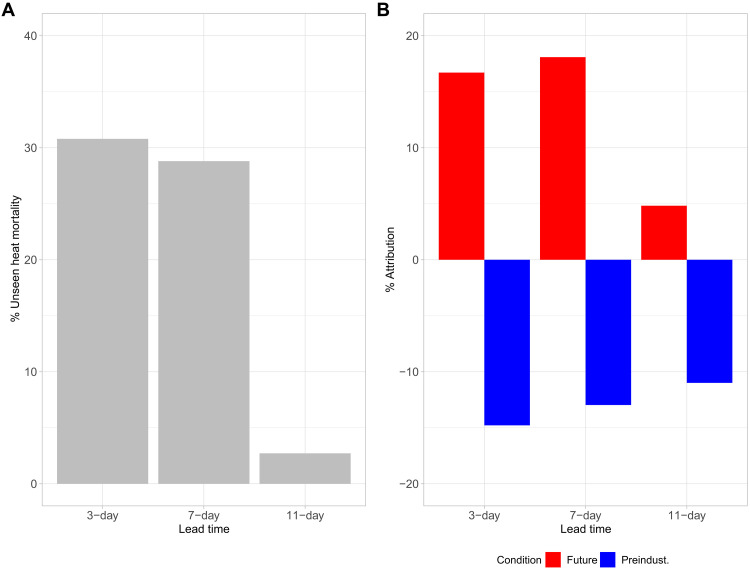
Percentage of unseen heatwave mortality and attribution for 3-, 7-, 11-day lead times in Vancouver. (**A**) Unseen heat-related mortality based on the “current” conditions. The percentage unseen heat-related mortality is calculated on the basis of the difference between average death from the three highest conditional exceedance probability and observed heat-related deaths divided by observed heat-related deaths. (**B**) Percentage of attributable mortality. The percentage attribution of future (red bars) is calculated as the difference in the number of heat-related deaths between “future” and “current” conditions divided by the sum of heat-related deaths under “current” conditions at the conditional exceedance probability where the “current” condition meets the observed heat-related deaths. The calculation of the percentage mortality attribution of preindustrial (blue bars) is the same but between “preindustrial” and “current” conditions.

In the worst cases (the three highest conditional exceedance probabilities under the “current” condition), there was a 1-in-17, 1-in-25.5, and 1-in-51 chance of seeing around 21 heat-related deaths in the 3-day lead time (Fig. 5A). That is equivalent to 31% more heat-related mortality than the observed number (= 16 deaths). We refer to this as “unseen” mortality, i.e., mortality that could have occurred but was not observed in real life. The percentage of unseen heat-related mortality is calculated on the basis of the difference between the average death from the three highest conditional exceedance probabilities and observed heat-related deaths divided by the observed heat-related deaths (21 − 16)/16 = 0.31 for the 3-day lead time. This is shown in Fig. 5A.

For the same heatwave event to occur in the future (at conditional exceedance probability, 1-in-1.2 chance, where the gray dots of “current” conditions crosses the dashed line of heat-related deaths from the observed temperature in Fig. 4), the number of heat-related mortality increases by 17% [= (18.7 − 16)/16]. For the preindustrial simulations, this number stands at 15% [= (13.66 − 16)/16] of heat-related deaths as attributable to anthropogenic climate change (Fig. 5B).

#### Seven-day lead time

For the 7-day lead time experiments, there is a 1-in-6.4, 1-in-2.7, and 1-in-1.7 chance of having the same number of heat mortality (16 deaths; black dashed line in Fig. 4, middle) as the PNW heatwave for “preindustrial,” “current,” and “future” scenarios, respectively. For the highest conditional exceedance probability in the “current” scenario, i.e., 1-in-17, 1-in-25.5, and 1-in-51 chance worst cases, we could have seen 29% more heat-related mortality than observed (Fig. 5A). For the same PNW heatwave event (exceedance probability at 2.7 for the “current” scenario), rises by 18% for the future scenario and drops by 13% for the preindustrial scenario when compared to heat-related deaths in current scenario (Fig. 5B).

#### Eleven-day lead time

When the forecast model is initialized 11 days before the heatwave, there is a 1-in-251, 1-in-83.7, and 1-in-50.2 chance to reach the same observed number of heat-related deaths during the PNW heatwave in “preindustrial,” “current,” and “future” scenarios, respectively. In the worst cases, there is a 1-in-251, 1-in-125.5, and 1-in-83.7 chance we could have seen, on average, 3% more heat-related deaths than observed (Fig. 5A). For the same heatwave event (conditional exceedance probability at 83.7 for “current”), there are 5% more heat-related deaths in future scenarios than in current, and 11% less under the preindustrial conditions (Fig. 5B).

#### Fraser East results

Fraser East HSDA (contains Abbotsford) gives very similar return periods to Vancouver HSDA, when compared to observed heat mortality (fig. S8). We note that the heat-related deaths are lower than in Vancouver, and as expected, daily heat-related deaths in Fraser East over 26 to 30 June 2021 are 22. In Fraser East, we could have seen 52, 48, and 20% more heat-related deaths than observed in 3-, 7-, and 11-day lead times (fig. S9A). For the same heatwave event to occur in the future, the number of heat-related deaths is increased by 17, 16 and 10% for 3-, 7-, and 11-day lead times, respectively (fig. S9B). For the preindustrial conditions, 15, 12, and 3% of heat-related deaths are attributable to anthropogenic climate change for 3-, 7-, and 11-day lead times, respectively (fig. S9B). The climate attribution of heat mortality was not performed in the other HSDAs as they did not demonstrate a relationship between the daily average temperature and all-cause mortality.

## DISCUSSION

We have demonstrated a framework of combining forecast-based attribution and an epidemiological model to attribute heat-related mortality during the unprecedented 2021 heatwave event in the PNW to human-induced climate change. This study shows the use of this attribution method in the field of health impact attribution. As forecast-based attribution reflects both storyline and probabilistic approaches, this allows us to have a range of attribution statements, with different framings, that can be used in different contexts depending on the audience. For example, policy-makers and health service providers might ask, “If a heatwave with similar physical drivers occurred again in a warmer future climate, what would the mortality impact be?” Under this framing, the 3-day lead time results (highly conditioned and closer to the storyline approach) would provide the most relevant attribution statement. On the other hand, the judge in a climate legal case might want to know “to what extent human-induced climate change has altered the mortality impact of a heatwave at least as severe as the 2021 PNW heatwave?” Under this framing, the 11-day lead time attribution statement that reflects a low level of conditioning and is closer to the probabilistic approach would be relevant. The use of a weather model rather than a climate model gives more reliable estimates of mortality during extreme events and is ideally suited to the health attribution community and the wider impact attribution communities. This is because weather forecast models have a longer and richer history of demand, scrutinization, and investment than climate models, often due to the serious socioeconomic implications of getting the weather forecast wrong (*29*, *30*).

By considering different lead times, we are able to understand and quantify how climate change affects processes that have different predictability horizons (effectively varying the level of conditioning) and the corresponding health impacts of the weather, while still using a model that demonstrably represents those processes adequately. If you are solely interested in the impact of climate change on the event in question exactly as it unfolded, then a short lead time is most appropriate as this is the most faithful representation of the event; this would be a canonical storyline framing of the attribution question. However, if the question is how the underlying risk of such an event is affected by climate change, then you need to consider how climate change is affecting the large-scale dynamics, and so you need to use a simulation in which those dynamics are (i) well represented, which is not necessarily the case in transient coarse (CMIP-type) climate model simulations, and (ii) able to respond to any perturbations made as part of the experiment design (which is not the case for short-lead forecasts). We do note that more work is needed on the interpretation and synthesis of results from different lead times, but this lies out of the scope of this study.

The heatwave attribution used in this study is simulations from the Integrated Forecast System, which is a high-resolution global forecast model; however, other weather models could have been used such as the Weather Research and Forecasting Model, a high-resolution limited-area model. However, full exploration of other weather forecast models lies outside of the scope of this study, which demonstrates a practical application of this approach.

In the epidemiological model, we have found that the RR of death increases with temperature in Vancouver and Fraser East HSDAs. In Vancouver HSDA, our framework has reliably predicted that very likely that the “future” climate has the highest heat-related mortality, then the “current” climate, and the lowest in the “preindustrial” climate at all three lead times. During the extreme heat event, we could have seen a 31 to 3% increase in heat-related mortality (i.e., unseen heatwave mortality under current climate conditions). In the likely occurrence of the PNW heatwave (prediction of “current” conditions matches the observed temperature in Fig. 4), 15 to 11% heat-related mortality is attributable to anthropogenic climate change. In the future without mitigation, an 18 to 5% increase can be seen in heat-related mortality compared to the observed in the PNW heatwave event.

We have found very different associations between temperature and mortality across the regions we considered; however, within-country variation in Canada was also previously identified (*14*). Now, no other studies undertake a heat-mortality event attribution in Canada, although one has taken a trend attribution approach globally, in which Canada is part of. In that, they estimated human-induced climate change heat-related mortality as the percentage of total deaths attributable to heat-related human-induced climate change, estimated as the difference in heat-related mortality between the factual and the counterfactual scenario in Canada (*14*) and found that 0.40, 0.21, and 0.48% of heat-related mortality was attributed to human-induced climate change in the city of Abbotsford, Victoria, and Vancouver, respectively. For comparison to our findings, Vancouver (more similar in areas covered in census metropolitan areas) estimated 5.0, 4.4, and 3.8% of total deaths attributable to heat-related human-induced climate change for 3-, 7-, and 11-day lead time, respectively. The only potential difference in result is that we were not able to replicate an effect between temperature and mortality in South Vancouver Island HSDA where Victoria is in; however, we note that their CI for human-induced climate change heat-related mortality in Victoria does include zero, which suggest that they may have also found lack of evidence of an effect between temperature and mortality, but it is difficult to know as the authors have not reported the RR. The crucial difference between our work and theirs is that they do a trend attribution over 1991 to 2018, whereas we do an event attribution of a specific heatwave. It is important to note that, although our approach focuses on a single extreme event, and the method can be generalized to any other past or future heatwave events, the quantitative assessment will differ depending on the specifics of that event. Relatedly, we show here that the mortality response between “the same” event in the future is not a linear increase from that of the past, but it is rather nonlinear for the longer lead times.

This study demonstrates how the forecast-based approach to attribution can be used for quantifying the anthropogenic influence on the health impacts of an extreme heatwave. It practically illustrates how the use of a forecast model–based approach reduces the need for statistical bias corrections and could pave the way for an operational impact attribution system. The “preindustrial” scenario meant that we could make statements of “what could have been without climate change” during the 2021 PNW heatwave. For example, if the same heatwave event has happened in 2021 without human-induced carbon emission, 15% of deaths could have been prevented. This quantitative statement can provide evidence for the developing world to make claims for loss and damages from climate change (*31*) and in climate litigations to force governments to change polices or large corporations to reduce their greenhouse gas emissions (*10*). The “future” scenario can also better prepare the local government by estimating mortality and estimating the financial cost of health facilities on the basis of policy-relevant targets. For example, if the temperature simulated in the counterfactual future forecast 7 days before is 2°C higher than usual summer temperatures, then it is very likely (1-in-5 chance) that there will be 28% more deaths than previous heatwaves. This probability gives policy-makers and health care providers an estimate of the risk and at what level of risk to balance the financial cost of health care and adverse health outcomes to the cost of increased preparedness and adaptation. Furthermore, we showed that other HSDAs did not have the same effect as seen in Vancouver and Fraser East; therefore, central BC health service can invest more on heat prevention policy in worse affected areas.

## MATERIALS AND METHODS

### Observational weather and health data

For modeling the exposure-response relationship, we used daily all-cause deaths count between 1981 and 2015 and observed daily mean temperatures for the same time period. Daily mortality was obtained from Statistics Canada. Daily mean air temperatures (in °C) were the average of 24-hourly measurements per day, obtained from the Daymet dataset. The Daymet dataset provided the daily temperature to a 1-km grid spatial resolution (*32*). Here, we focused on British Columbia’s HSDAs (*33*) that contained major cities or villages affected in the PNW heatwave. These were the South Vancouver Island health region (including the city of Victoria), Vancouver (including Vancouver), the Fraser East health region (including Abbotsford), and the Thompson/Cariboo health region (including the village of Lytton). Our mortality data represented the total daily mortality within each of these regions. The daily average temperature was obtained using a population-weighted average of all 1-km grids from the Daymet data (*32*). We restricted the data to summer months (June to September) for the analysis. Table 1 shows the total and average daily deaths, as well as average summer temperature, in these HSDAs during the data period (1981 to 2015).

### Modeling experiments

We use the PNW heatwave attribution data generated in (*21*). In the main text, we have highlighted the reasons why forecast-based attribution has been used to predict heat-related mortality and will summarize here why this type of attribution as a function of forecast time horizon is an advantage in the perspective of climate attribution. Forecast-based attribution could combine two “competing” attribution frameworks: the storyline (highly conditioned) and probabilistic (“unconditioned”) approaches. This will likely be critical for events where the two frameworks disagree, especially important as both frameworks are used to quantify the anthropogenic influence on the socioeconomic impacts of the weather. The intention of the weather forecast model is to predict the weather, and therefore it is possible to test whether the model is able to forecast the event in question (and for the relevant underlying physical processes). This provides a “suitability test” of the model for understanding the impact of climate change on the extreme event of interest. There are other reasons mentioned in Introduction: the potential for operational and impact attribution, an improved understanding of climate change impacts on atmospheric circulation using models that demonstrably represent those dynamics faithfully, a bias correction–free approach, and its reliability of the weather model due to its long history and detrimental consequence if the model fails.

The reader is referred to a study for full experimental design details (*21*). In brief, these data were created by replicating the ECMWF operational ensemble forecast system and perturbing the initial and boundary conditions of the model to produce counterfactual climate forecasts. We introduced hydrostatically balanced perturbations to the 3D ocean temperature and salinity fields and modified the atmospheric CO_2_ concentrations. We generated the perturbations by estimating the anthropogenic fingerprint since the preindustrial period in surface and subsurface observations of the ocean. We extracted the 3-, 7-, and 11-day lead times, ending in the heatwave peak period of 2021 (28 to 30 June). Because of computational time, only the 11-day lead time has 251 ensemble members, and the other lead times have 51 ensemble members.

The authors in heatwave attribution data (*21*) noted that the local response varies with global average surface temperature response depending on lead times and scenarios; therefore, different scaling factors are required to adjust for this difference in “preindustrial” and “future” scenarios. We have used the following steps:

1) Calculate daily average surface temperature anomalies between future and preindustrial scenarios for local and global response.

2) Average anomalies across the ensembles for local and global response.

3) Extract coefficient from the linear regression of global average temperature response on local response for 26 and 27 June.

4) The scaling factor is then calculated using the following formula

$$\begin{array}{c} \left(1.\;6\;-\;\text{estimated global average temperature response}\right)*\bar{\beta } \end{array}$$

1.6 is the present-day observed attributable to global mean land warming (*34*). $\bar{\beta }$ is the average coefficient of 26 and 27 June from step (3).

5) The scaling factor is then subtracted and added to the local average temperature response of “preindustrial” and “future” scenario, respectively.

For the daily average temperature to be compatible with temperature values in the health data, the coordinates for the local responses of each HSDA are based on coordinates that is closest to the population-weighted centroid *x* and *y* coordinates from the health data. This step was implemented in JASMIN, the UK’s collaborative data analysis environment (https://www.jasmin.ac.uk).

### Modeling the exposure-response relationship

Using distributed lag nonlinear models (DLNMs) to model the delayed and nonlinear relationship between the daily mean temperature and all-cause mortality (*35*)

$$\text{log}\left[E\left(Y_{t}\right)\right]=\alpha +f\left(x_{t};\theta \right)+\sum_{k=1}^{2}\gamma_{k}u_{tk}$$ (1)

Our outcome $Y_{t}$ will be death counts at day *t*, assumed to follow a Poisson distribution with overdispersion. $x_{t}$ is mean the temperature, and $z_{t}$ is days of the week at day *t*. Function $f\left(x_{t};\theta \right)$ specifies the association with $x_{t}$ , i.e., mean temperature *x* at day *t*, through a bidimensional cross-basis term, using flexible natural cubic spline functions to model both exposure-response and lagged-response dimensions, accounting for 3 days of lag. The exposure-response dimension is cubic splines with two knots (for 50th and 98th percentiles of the temperature distribution). The model includes confounders, $u_{tk}$ , days of the week, and natural cubic splines of time with four degrees per year to control for long-term trends and seasonal effects. The choice of DLNMs to model the exposure-response relationship and variables within has been previously detailed in the peer-reviewed literature (*14*, *35*, *36*).

For sensitivity analysis, we fit the above DLNM to data that are stratified by age (under 65 years old, between ages 65 and 74, and over 74 years old) and sex (male or female).

### Predicting mortality with temperatures

We use the following to calculate daily attributable deaths, $D_{\text{HW}}$ , due to heat (*35*)

$$D_{\text{HW}}=D_{E}\left\{1-e^{-\left[f^{*}\left(T_{\text{fore}};\theta^{*}\right)-s^{*}\left(T_{\text{MM}};\theta^{*}\right)\right]}\right\}$$ (2)

where $D_{E}$ is the expected daily deaths counts, which is calculated by averaging daily mortality across 1981 to 2015 (for Vancouver HSDA, we only used data after the boundary change, i.e., between 2008 and 2015), excluding leap days. $T_{\text{fore}}$ and $T_{\text{MM}}$ are the daily mean temperature from the heatwave attribution data and the minimum mortality temperature, respectively. $f^{*}$, $s^{*}$ , and $\theta^{*}$ represent the unidimensional overall cumulative exposure-response curves with reduced lag dimension, derived from the bidimensional term estimated by Eq. 1.

To account for the uncertainty around the estimation of exposure-response association, we have used Monte Carlo simulation of 1000 samples, sampling at random multivariate normal distribution with the mean of estimated DLNM coefficients and variance is the estimated covariance matrix.

All analyses were implemented using R (v4.4.3). The DLNM model was performed using dlnm R package (v2.4.7).

### Contextualization of predicted heat-related mortality

We used ERA5 analysis (*28*) as observed events to compare it to the predicted heat-related mortality from attribution temperature values. We extracted the daily average temperature from the closest coordinates to the population-weighted centroid *x* and *y* coordinates in the health data.

The conditional exceedance probability of ensemble member *i* is calculated as the total number of ensemble members divided by rank (-1*sum of deaths over the heatwave period of *i*).

## Supplementary Material

20251119-1

## Supplementary Materials

### This PDF file includes:

Supplementary Text Figs. S1 to S9 Table S1 References

Erratum published 7 August 2026:
Figures 1, 4, and 5, and figs. S8 and S9 have been corrected to reflect the lowering of the values from the “future” scenario. The authors’ conclusions are not affected by these corrections. The online and PDF versions of the article and the Supplementary Materials PDF have been corrected. The original version of the the Supplementary Materials is available here:
