# Supplementary material for: Forecast attribution reveals enhanced heat mortality from climate change in British Columbia heatwave
Source: Sci Adv. 2025 Nov 19;11(47):eadw8268. doi: 10.1126/sciadv.adw8268 (PMC12629175; doi:10.1126/sciadv.adw8268)
Supplement: Supplementary file 1 — Supplementary Text Figs. S1 to S9 Table S1 References [file sciadv.adw8268_sm.pdf]

Science Advances
AAAS

Supplementary Materials for

Forecast attribution reveals enhanced heat mortality from climate change in British Columbia heatwave

Chin Yang Shapland *et al.*

Corresponding author: Chin Yang Shapland, chinyang.shapland@bristol.ac.uk

Sci. Adv. **11**, eadw8268 (2025)
DOI: 10.1126/sciadv.adw8268

This PDF file includes:

Supplementary Text
Figs. S1 to S9
Table S1
References

Supplementary Text

Weather and climate models

The distinguishing features between "climate" and "weather" model; "weather" model gives short term atmospheric changes and is dependent on initial conditions, i.e. whether a storm is predicted tomorrow, is dependent on the weather conditions today and model dynamics. "Weather" models generally reliably predict the weather up to 15 days ahead. "Climate" model determines long-term (usually 30-years) weather statistics, it projects the average or extreme weather at a specific region and timeline, and probability of extremes. "Climate" model output is dependent on the boundary conditions of and parameters in the models rather than the initial conditions (*37*).

Fig. S1.

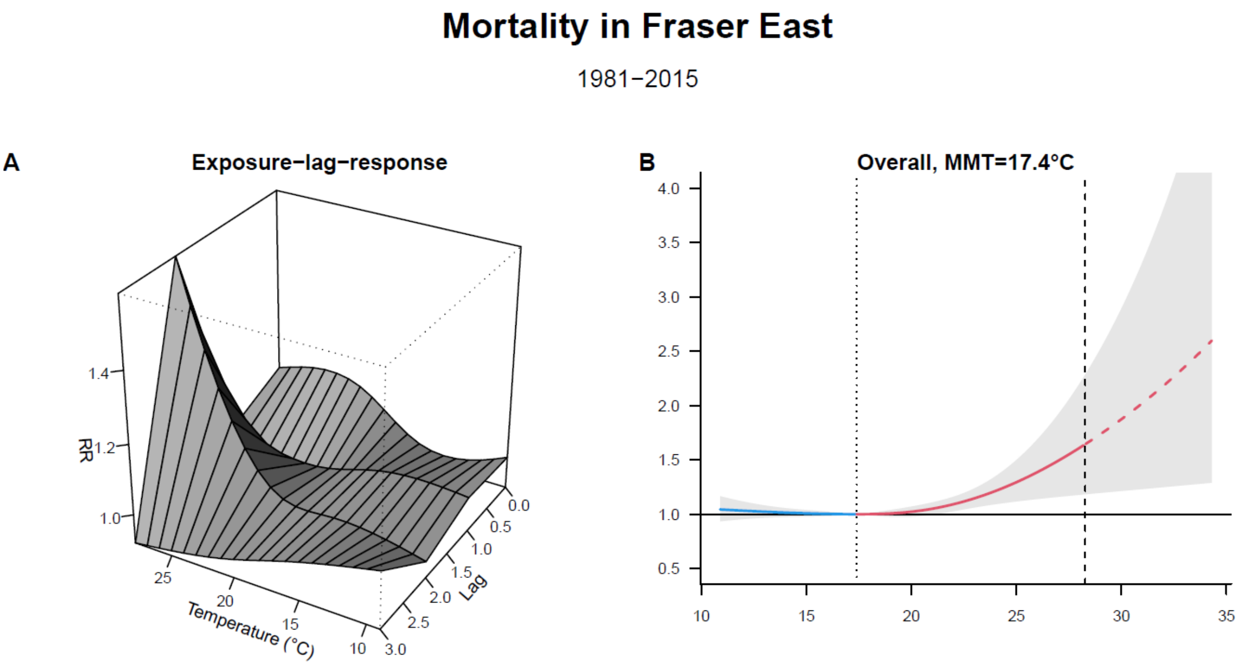

Temperature-related mortality in Fraser East (1981-2015). (A) is the three-dimensional plot showing the estimated exposure-lag-response association between temperature and mortality, where y-axis, x-axis and z-axis is relative risk (RR), daily average temperature (in Celsius) and lag time (in days), respectively. (B) plot showing overall cumulative mortality risk, y-axis is the RR and x-axis is daily average temperature in Celsius. Dotted line is the minimum mortality temperature (MMT).

Fig. S2

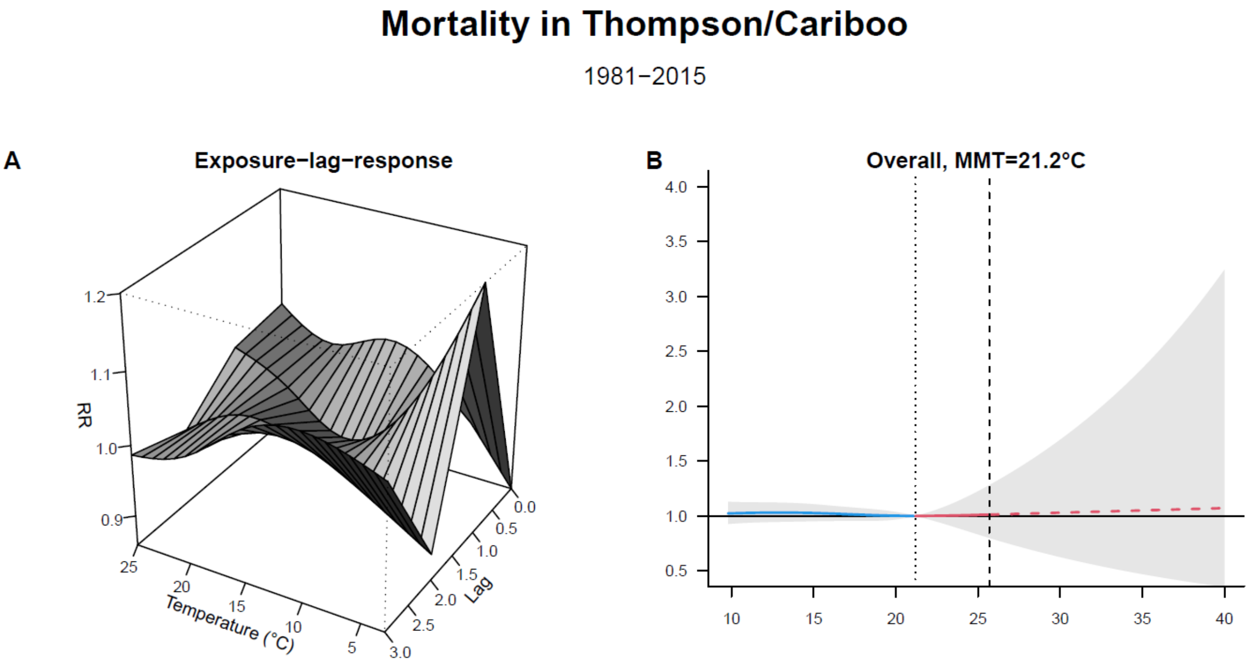

Temperature-related mortality in Thompson/Cariboo (1981-2015). (A) is the three-dimensional plot showing the estimated exposure-lag-response association between temperature and mortality, where y-axis, x-axis and z-axis is relative risk (RR), daily average temperature (in Celsius) and lag time (in days), respectively. (B) plot showing overall cumulative mortality risk, y-axis is the RR and x-axis is daily average temperature in Celsius. Dotted line is the minimum mortality temperature (MMT).

Fig. S3.

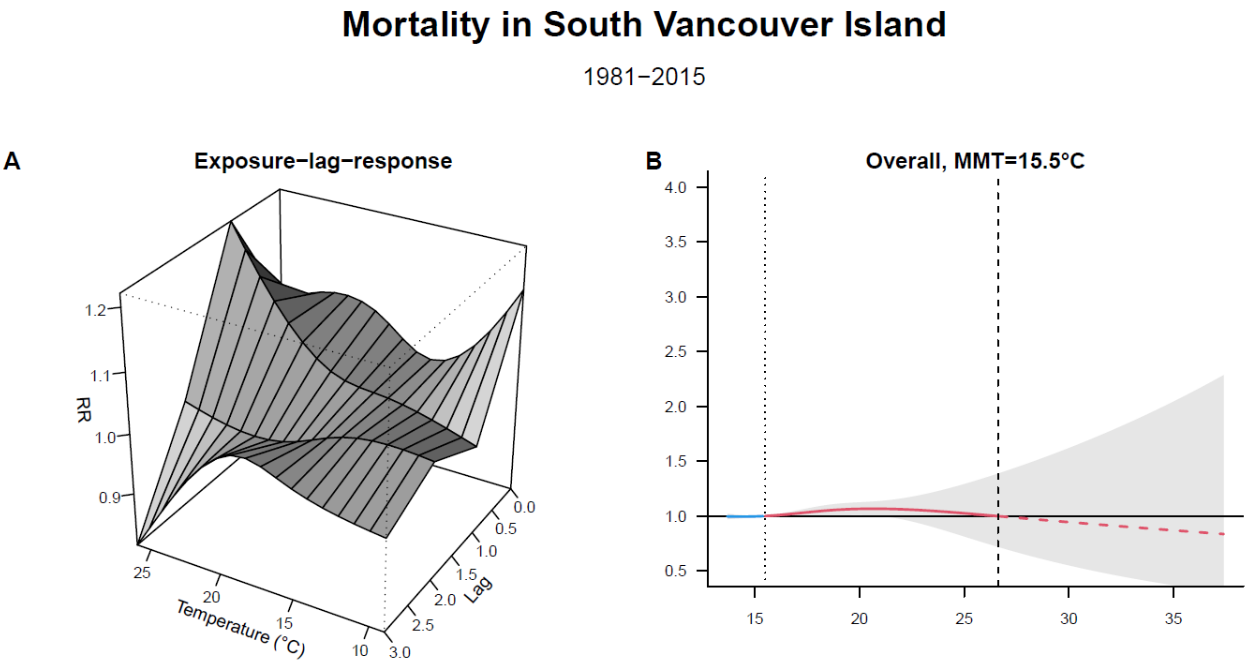

Temperature-related mortality in South Vancouver Island (1981-2015). (A) is the three-dimensional plot showing the estimated exposure-lag-response association between temperature and mortality, where y-axis, x-axis and z-axis is relative risk (RR), daily average temperature (in Celsius) and lag time (in days), respectively. (B) plot showing overall cumulative mortality risk, y-axis is the RR and x-axis is daily average temperature in Celsius. Dotted line is the minimum mortality temperature (MMT).

Fig. S4.

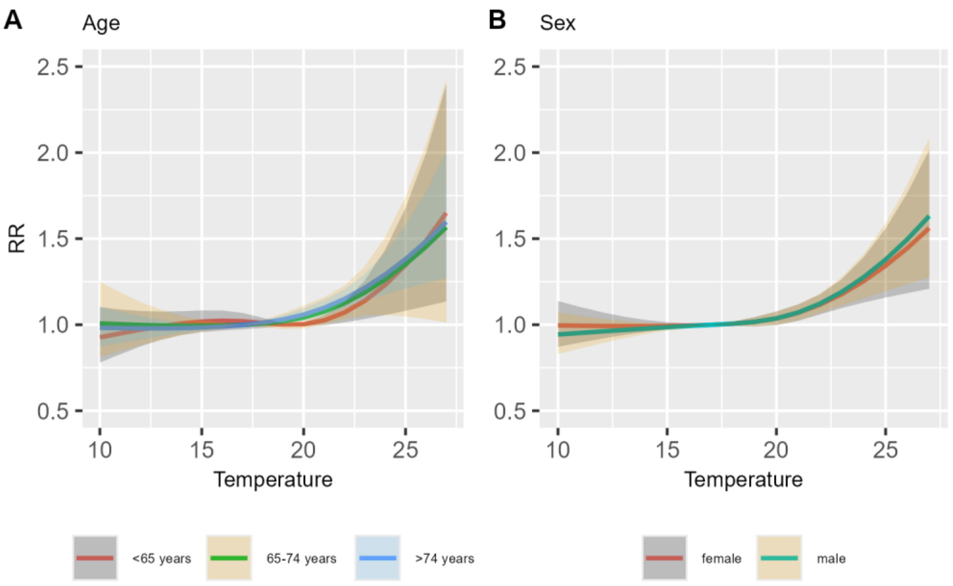

Vancouver (A) Age-specific exposure-response curve. (B) Sex-specific exposure-response curve.

Fig. S5.

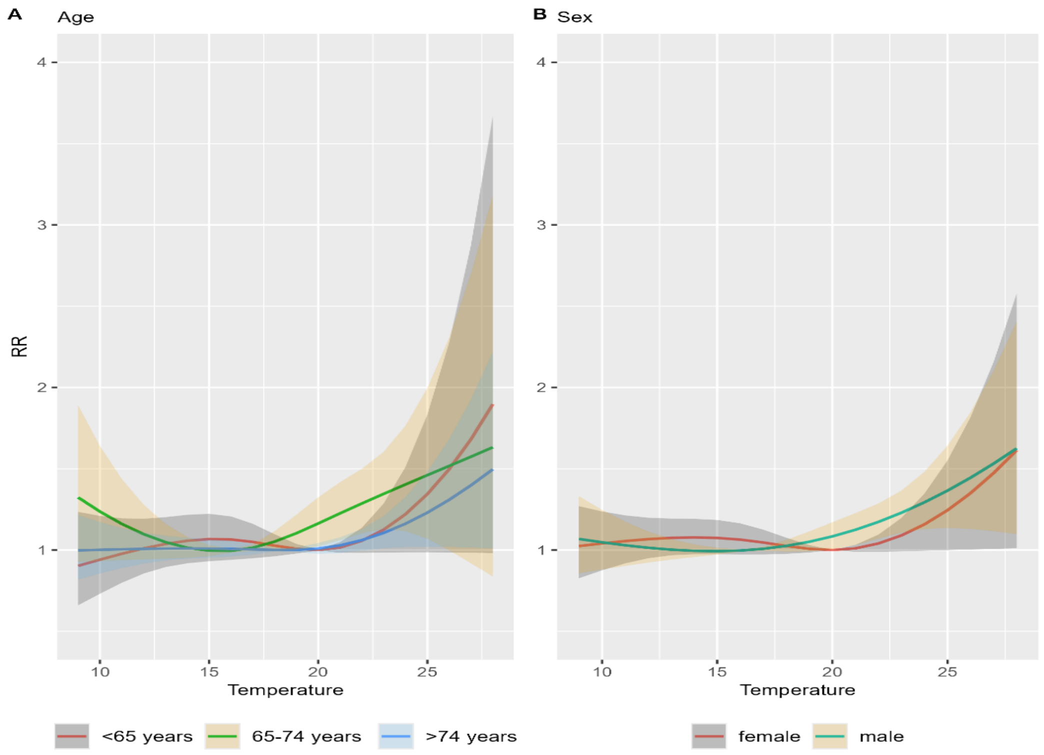

Fraser East. A, Age-specific exposure-response curve. B, Sex-specific exposure-response curve.

Fig. S6.

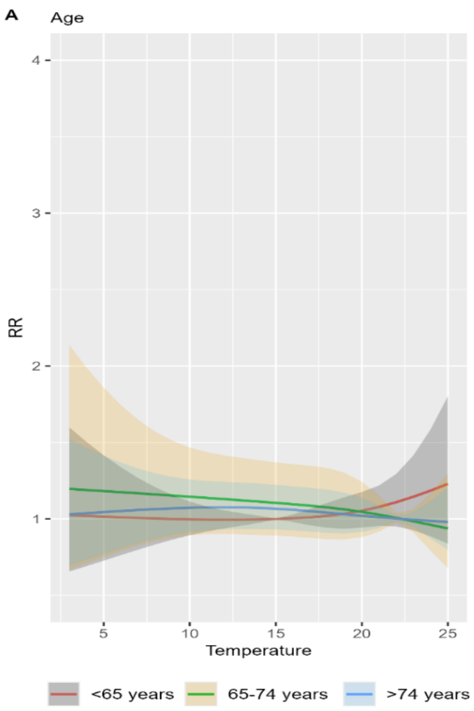

Thompson/Cariboo. A, Age-specific exposure-response curve. B, Sex-specific exposure-response curve.

Fig. S7.

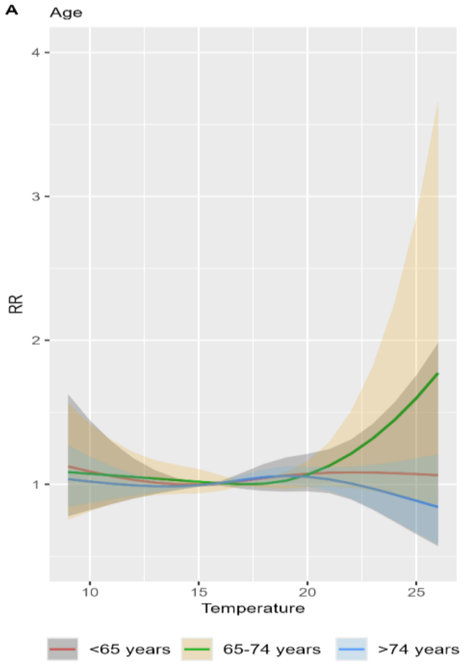

South Vancouver Island. A, Age-specific exposure-response curve. B, Sex-specific exposure-response curve.

Fig. S8.

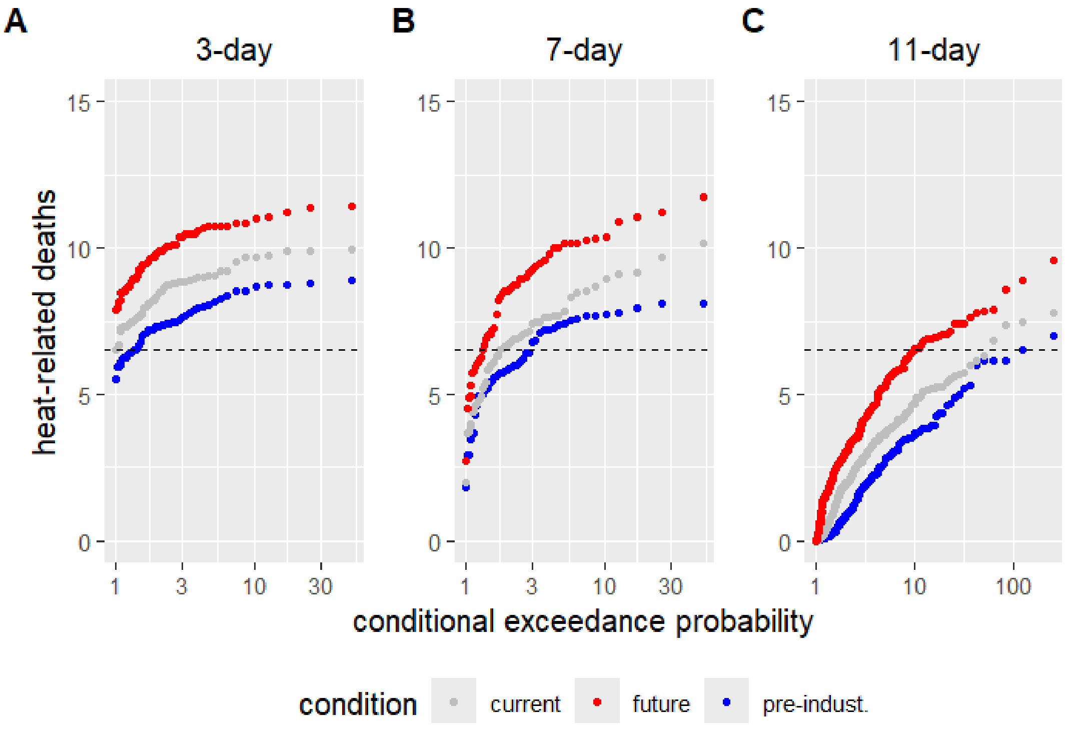

Conditional exceedance probability for heat-related deaths during the PNW heatwave predicted from operational and counterfactual forecast ensembles in Fraser East HDSA. The y-axis is the sum of heat-related deaths across 26th-30th of June 2021 (i.e. PNW heatwave). The x-axis is log-scaled. Each panel shows ensembles initialized at the lead given above the panel. Red, grey and blue dots indicate empirical return-time plots for heat-related mortality predicted from daily mean temperatures of the ensemble members of the future, current and pre-industrial forecasts. The black dashed line shows heat-related deaths based on the observed average temperature (ERA5 reanalysis) during the PNW heatwave.

Fig. S9.

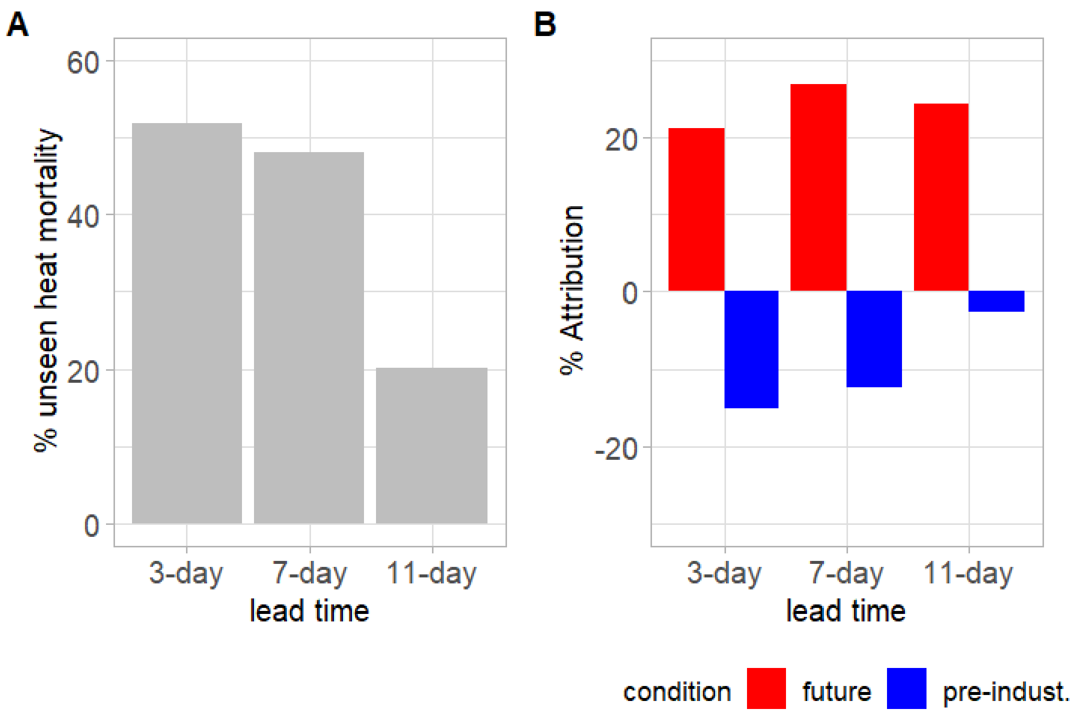

Percentage of unseen and attribution for 3-,7-, 11-day lead time in Fraser East. (A) gives the unseen heat-related mortality based on the "current" conditions. The percentage unseen heat-related mortality is calculated based on the difference between average death from three highest conditional probability and observed heat-related deaths, divided by observed heat-related deaths. (B) gives the percentage of heat-related deaths as attributable to anthropogenic climate change for the same PNW 2021 heatwave event in Vancouver. The percentage attribution of future (red bars) is calculated as the difference in number of heat-related deaths between "future" and "current" conditions divided by sum of heat-related deaths in "current" conditions, at the conditional exceedance probability where "current" condition crosses the observed heat-related deaths. The calculation of percentage attribution of pre-industrial (blue bars) is the same but between "pre-industrial" and "current" conditions.

Table S1.

Summary of observed temperature and all-cause mortality, in female and over age 65 years, over summer period (June-September) in each Health Service Delivery Area.

City	*Data period*	*Total deaths*	*Percentage of total deaths in female (%)*	*Percentage of total deaths in between age 65-75 years (%)*	*Percentage of total deaths in over age 75 years (%)*
Fraser East	1981-2015	18,275	46	19	57
Thompson/Cariboo	1981-2015	15,334	42	21	46
Vancouver	1991-2007	67,818	48	18	57
Vancouver*	2008-2015	9,375	48	18	57
South Vancouver Island	1981-2015	34,423	51	17	46

*The health boundaries of Vancouver have changed in 2008. SD is the standard deviation.

REFERENCES AND NOTES

1. K. L. Ebi, A. Capon, P. Berry, C. Broderick, R. de Dear, G. Havenith, Y. Honda, R. S. Kovats, W. Ma, A. Malik, N. B. Morris, L. Nybo, S. I. Seneviratne, J. Vanos, O. Jay, Hot weather and heat extremes: Health risks. *Lancet* **398**, 698–708 (2021).

2. Y. E. Lo, E. Vosper, J. P. Higgins, G. Howard, Heat impacts on human health in the Western Pacific Region: An umbrella review. *Lancet Reg. Health West Pac.* **42**, 100952 (2024).

3. D. Mitchell, Y. T. E. Lo, E. Ball, J. L. Godwin, O. Andrews, R. Barciela, L. B. Ford, C. D. Napoli, K. L. Ebi, N. S. Fučkar, A. Gasparrini, B. Golding, C. L. Gregson, G. J. Griffith, S. Khalid, C. Robinson, D. N. Schmidt, C. H. Simpson, R. S. J. Sparks, J. G. Walker, Expert judgement reveals current and emerging UK climate-mortality burden. *Lancet Planet Health* **8**, e684–e694 (2024).

4. D. Mitchell, Why we still don't know the mounting health risks of climate change. *Nature* **637**, 766 (2025).

5. V. Thompson, D. Mitchell, G. C. Hegerl, M. Collins, N. J. Leach, J. M. Slingo, The most at-risk regions in the world for high-impact heatwaves. *Nat. Commun.* **14**, 2152 (2023).

6. V. Thompson, A. T. Kennedy-Asser, E. Vosper, Y. T. E. Lo, C. Huntingford, O. Andrews, M. Collins, G. C. Hegerl, D. Mitchell, The 2021 western North America heat wave among the most extreme events ever recorded globally. *Sci. Adv.* **8**, eabm6860 (2022).

7. British Columbia Coroners Service, "Extreme heat and human mortality: A review of heat-related deaths in B.C. in summer 2021" (2022); https://www2.gov.bc.ca/assets/gov/birth-adoption-death-marriage-and-divorce/deaths/coroners-service/death-review-panel/extreme_heat_death_review_panel_report.pdf.

8. D. G. Clark, E. H. Jackson, C. M. Hohl, K. E. Liang, Extreme heat impacts on acute care: Examining emergency department visits and hospital admissions during the 2021 British Columbia heatwave. *J. Clin. Chang. Health* **17**, 100310 (2024).

9. D. Mitchell, C. Heaviside, N. Schaller, M. Allen, K. L. Ebi, E. M. Fischer, A. Gasparrini, L. Harrington, V. Kharin, H. Shiogama, Extreme heat-related mortality avoided under Paris Agreement goals. *Nat. Clim. Chang.* **8**, 551–553 (2018).

10. R. F. Stuart-Smith, F. E. Otto, A. I. Saad, G. Lisi, P. Minnerop, K. C. Lauta, K. Van Zwieten, T. Wetzer, Filling the evidentiary gap in climate litigation. *Nat. Clim. Chang.* **11**, 651–655 (2021).

11. C. J. Carlson, D. Mitchell, R. Gibb, R. F. Stuart-Smith, T. A. Carleton, T. E. Lavelle, C. J. Lippi, M. Lukas-Sithole, M. A. North, S. J. Ryan, Health losses attributable to anthropogenic climate change. medRxiv 2024.08.07.24311640 [Preprint] (2024). https://doi.org/10.1101/2024.08.07.24311640.

12. R. Stuart-Smith, A. Vicedo-Cabrera, S. Li, F. Otto, K. Belesova, A. Haines, L. Harrington, J. Hess, R. Venkatraman, T. Wetzer, A. Woodward, K. Ebi, Quantifying heat-related mortality attributable to human-induced climate change (2023); https://doi.org/10.21203/rs.3.rs-2702337/v1.

13. D. Mitchell, C. Heaviside, S. Vardoulakis, C. Huntingford, G. Masato, B. P. Guillod, P. Frumhoff, A. Bowery, D. Wallom, M. Allen, Attributing human mortality during extreme heat waves to anthropogenic climate change. *Environ. Res. Lett.* **11**, 074006 (2016).

14. A. M. Vicedo-Cabrera, N. Scovronick, F. Sera, D. Royé, R. Schneider, A. Tobias, C. Astrom, Y. Guo, Y. Honda, D. Hondula, The burden of heat-related mortality attributable to recent human-induced climate change. *Nat. Clim. Chang.* **11**, 492–500 (2021).

15. T. M. Beck, D. L. Schumacher, H. Achebak, A. M. Vicedo-Cabrera, S. I. Seneviratne, J. Ballester, Mortality burden attributed to anthropogenic warming during Europe's 2022 record-breaking summer. *NPJ Clim. Atmos. Sci.* **7**, 245 (2024).

16. E. Bercos-Hickey, T. A. O'Brien, M. F. Wehner, L. Zhang, C. M. Patricola, H. Huang, M. D. Risser, Anthropogenic contributions to the 2021 Pacific Northwest heatwave. *Geophys. Res. Lett.* **49**, e2022GL099396 (2022).

17. S. Y. Philip, S. F. Kew, G. J. Van Oldenborgh, F. S. Anslow, S. I. Seneviratne, R. Vautard, D. Coumou, K. L. Ebi, J. Arrighi, R. Singh, Rapid attribution analysis of the extraordinary heat wave on the Pacific coast of the US and Canada in June 2021. *Earth Syst. Dynam.* **13**, 1689–1713 (2022).

18. K. A. McKinnon, I. R. Simpson, How unexpected was the 2021 Pacific Northwest heatwave? *Geophys. Res. Lett.* **49**, e2022GL100380 (2022).

19. L. Zhang, M. D. Risser, M. F. Wehner, T. A. O'Brien, Leveraging extremal dependence to better characterize the 2021 Pacific Northwest heatwave. *J. Agric. Biol. Environ. Stat.* **2024**, 10.1007/s13253-024-00636-8 (2024).

20. R. Emerton, C. Brimicombe, L. Magnusson, C. Roberts, C. Di Napoli, H. L. Cloke, F. Pappenberger, Predicting the unprecedented: Forecasting the June 2021 Pacific Northwest heatwave. *Weather* **77**, 272–279 (2022).

21. N. J. Leach, C. D. Roberts, M. Aengenheyster, D. Heathcote, D. M. Mitchell, V. Thompson, T. Palmer, A. Weisheimer, M. R. Allen, Heatwave attribution based on reliable operational weather forecasts. *Nat. Commun.* **15**, 4530 (2024).

22. H. Lin, R. Mo, F. Vitart, The 2021 western North American heatwave and its subseasonal predictions. *Geophys. Res. Lett.* **49**, e2021GL097036 (2022).

23. R. Mo, H. Lin, F. Vitart, An anomalous warm-season trans-Pacific atmospheric river linked to the 2021 western North America heatwave. *Commun. Earth Environ.* **3**, 127 (2022).

24. G. Zappa, T. G. Shepherd, Storylines of atmospheric circulation change for European regional climate impact assessment. *J. Clim.* **30**, 6561–6577 (2017).

25. A. D. King, G. J. van Oldenborgh, D. J. Karoly, S. C. Lewis, H. Cullen, Attribution of the record high Central England temperature of 2014 to anthropogenic influences. *Environ. Res. Lett.* **10**, 054002 (2015).

26. N. Massey, R. Jones, F. E. L. Otto, T. Aina, S. Wilson, J. M. Murphy, D. Hassell, Y. H. Yamazaki, M. R. Allen, weather@home—Development and validation of a very large

ensemble modelling system for probabilistic event attribution. *Q. J. R. Meteorol. Soc.* **141**, 1528–1545 (2015).

27. N. J. Leach, S. Li, S. Sparrow, G. J. van Oldenborgh, F. C. Lott, A. Weisheimer, M. R. Allen, Anthropogenic influence on the 2018 summer warm spell in Europe: The impact of different spatio-temporal scales. *Bull. Am. Meteorol. Soc.* **101**, S41–S46 (2020).

28. H. Hersbach, B. Bell, P. Berrisford, S. Hirahara, A. Horányi, J. Muñoz-Sabater, J. Nicolas, C. Peubey, R. Radu, D. Schepers, A. Simmons, C. Soci, S. Abdalla, X. Abellan, G. Balsamo, P. Bechtold, G. Biavati, J. Bidlot, M. Bonavita, G. De Chiara, P. Dahlgren, D. Dee, M. Diamantakis, R. Dragani, J. Flemming, R. Forbes, M. Fuentes, A. Geer, L. Haimberger, S. Healy, R. J. Hogan, E. Hólm, M. Janisková, S. Keeley, P. Laloyaux, P. Lopez, C. Lupu, G. Radnoti, P. de Rosnay, I. Rozum, F. Vamborg, S. Villaume, J.-N. Thépaut, The ERA5 global reanalysis. *Q. J. R. Meteorol. Soc.* **146**, 1999–2049 (2020).

29. S. Bordoni, S. M. Kang, T. A. Shaw, I. R. Simpson, L. Zanna, The futures of climate modeling. *NPJ Clim. Atmos. Sci.* **8**, 99 (2025).

30. G. Brunet, D. B. Parsons, D. Ivanov, B. Lee, P. Bauer, N. B. Bernier, V. Bouchet, A. Brown, A. Busalacchi, G. C. Flatter, R. Goffer, P. Davies, B. Ebert, K. Gutbrod, S. Hong, P. K. Kenabatho, H.-J. Koppert, D. Lesolle, A. H. Lynch, J.-F. Mahfouf, L. Ogallo, T. Palmer, K. Petty, D. Schulze, T. G. Shepherd, T. F. Stocker, A. Thorpe, R. Yu, Advancing weather and climate forecasting for our changing world. *Bull. Am. Meteorol. Soc.* **104**, E909–E927 (2023).

31. I. Noy, M. Wehner, D. Stone, S. Rosier, D. Frame, K. A. Lawal, R. Newman, Event attribution is ready to inform loss and damage negotiations. *Nat. Clim. Chang.* **13**, 1279–1281 (2023).

32. P. E. Thornton, R. Shrestha, M. Thornton, S.-C. Kao, Y. Wei, B. E. Wilson, Gridded daily weather data for North America with comprehensive uncertainty quantification. *Sci. Data* **8**, 190 (2021).

33. Government of British Columbia, British Columbia's Health Boundary Maps (2025); https://www2.gov.bc.ca/gov/content/health/about-bc-s-health-care-system/partners/health-authorities/health-boundary-maps.

34. T. J. Osborn, P. D. Jones, D. H. Lister, C. P. Morice, I. R. Simpson, J. P. Winn, E. Hogan, I. C. Harris, Land surface air temperature variations across the globe updated to 2019: The CRUTEM5 data set. *J. Geophys. Res. Atmos.* **126**, e2019JD032352 (2021).

35. A. M. Vicedo-Cabrera, F. Sera, A. Gasparrini, Hands-on tutorial on a modeling framework for projections of climate change impacts on health. *Epidemiology* **30**, 321–329 (2019).

36. J. Boudreault, C. Campagna, É. Lavigne, F. Chebana, Projecting the overall heat-related health burden and associated economic costs in a climate change context in Quebec, Canada. *Sci. Total Environ.* **958**, 178022 (2025).

37. J. T. Houghton, Y. Ding, D. J. Griggs, M. Noguer, P. J. van der Linden, X. Dai, K. Maskell, C. A. Johnson, "Climate Change 2001: The Scientific Basis. Contribution of Working Group I to the Third Assessment Report of the Intergovernmental Panel on Climate Change" (Cambridge Univ. Press, 2001).
